# A prospective study of erectile dysfunction in men after pelvic surgical procedures and its association with non-modifiable risk factors

**DOI:** 10.1186/s13104-019-4839-2

**Published:** 2019-12-18

**Authors:** S. Artemi, P. Vassiliu, N. Arkadopoulos, Maria-Eleni Smyrnioti, P. Sarafis, V. Smyrniotis

**Affiliations:** 1 Department of Nursing, General Hospital of Athens “ELPIS”, Athens University of Technology, Athens, Greece; 2 School of Medicine, National and Kapodistrian University of Athens, Athens, Greece; 3 Freelance Systemic Family Therapist, Athens, Greece; 4 Department of Nursing, Cyprus University of Technology, 30 Archbishop Street, 3036 Limassol, Cyprus

**Keywords:** Erectile dysfunction, Pelvic surgery, Risk factors

## Abstract

**Objective:**

A pelvic surgery can cause erectile dysfunction. The purpose of this study was to evaluate erectile function at various times after pelvic surgery in male patients; to search the non-modifiable risk factors associated with the presence and intensity of sexuality in these patients. This prospective study used the erectile dysfunction IIEF scale.

**Results:**

The study population comprised of 106 male patients who had undergone minor pelvic surgery at least 9 months before and during the 2010–2016 period in the 4th Surgical Clinic. A control group of healthy males (N = 106) who underwent no pelvic surgery matched for age was also used for reference values. The main age of the participants was 66.16 ± 13.07 years old. A history of colectomy was present in 36.8%, 18.9% had undergone sigmoidectomy, and 33% inguinal hernia repair. The percentage of severe erectile function increased from 38.7% before surgery to 48.1% (25% increase) after surgery, at the end of the follow-up period (p < 0.05). In the multivariate analysis model, age emerged as an independent predictor of erectile function (p < 0.001). Age was the most important determinant of the IIEF score, which was aggravated by 25% from the first to the last assessment of patients.

## Introduction

Erectile dysfunction (ED) is defined as the inability to achieve and maintain an erection sufficient for satisfactory sexual intercourse [1]. Many causal factors have been implicated. Indeed, erectile dysfunction can arise from vascular or neurogenic cause or be associated with anatomical and hormonal disorders, psychogenic or drug related [2, 3].

Erectile dysfunction can also be attributed to pelvic surgery, because of either vascular or neurogenic factors alone, or a combination of both. Pelvic surgical procedures in males, which are associated with significant erectile dysfunction, include radical prostatectomy (RP), radical cystectomy and low anterior or abdominoperineal resection (APRs) for rectal cancer [4].

Erectile function recovery rates vary widely in different studies, depending on the type of surgery, surgeon’s surgical technique and experience, ranging between 20 and 80%. Age also appears to have a significant effect on the restoration of erectile function, as there is a significant reduction in strength with increasing age, with patients aged less than 60 years achieving up to 50% higher rates compared to patients over the age of 60 [5, 6].

The purpose of the study was to evaluate erectile function at various time points after pelvic surgery in male patients and to investigate the non-modifiable risk factors associated with the presence and intensity of sexuality in these patients.

## Main text

### Methods

#### Participants and procedure

This was a prospective study conducted in the University Hospital “ATTIKON”. The study population consisted of all male patients who had undergone pelvic surgery at least 9 months before and during the 2010–2016 period in the 4th Surgical Clinic. A control group (subjects with no pelvic surgery and serious comorbidities) matched for age was also included in the study, providing IIEF reference values for this age group.

*Exclusion criteria* from the study included disorder of consciousness, dementia, diagnosed mental illness, and objective communication failure (deafness or blindness, not speaking the Greek language). The study involved 106 patients.

#### Data collection

Patients’ demographic and clinical characteristics were recorded, and then the severity of the problem was depicted on the International Index of Erectile Scale function (IIEF) as well as any changes observed in patients’ quality of life. Patients were evaluated prior to surgery (Phase 1) as well as 3 (Phase 2), 6 (Phase 3), and 9 (Phase 4) months after surgery with the IIEF scale.

#### Measures

The data were collected by interview using a structured questionnaire consisting of four parts:

1. Demographic (sex, age, occupation, family and financial status) and clinical features (medical history).
2. IIEF (International Index of Erectile Function) scale [7].

The patients completed the questionnaire in the presence of the investigator at the hospital, during their regular follow-up. This was feasible for patients living in Attica and some from the province. The rest of the sample filled out the questionnaires by telephone.

#### Statistical analysis

Data processing was performed with SPSS 22.0. Descriptive and inferential statistics was executed. The parametric t- test, (for comparisons between controls and patients and between patient subgroups), repeated measures analysis for consecutive measures at the different time points, and multiple linear regression models (for erectile function prediction) were applied. Control/patients group was entered as a dummy variable along with age in regression model. Statistical significance level was set at p < 0.05.

### Results

A 75% of the participants were married and 65.1% retired. Primary School graduates were 34% and Junior High School 34.9%. University graduates or were 4.7%. The mean age of the participants was 66.16 ± 13.07 years old. Regarding comorbidity, 47.2% reported various cardiovascular problems, 20.8% hypertension, 9.4% diabetes mellitus and 5.7% depression. A history of colectomy was present in 36.8%, 18.9% had undergone sigmoidectomy, and 33% inguinal hernia repair. Control group mean age was 63.95 ± 9.91 years old vs 66.16 ± 13.06 years old for study group (p = 0.167). Before surgery, radiotherapy was administered to 25.5% of patients, while 8.5% had undergone chemotherapy and 0.9% hormonal therapy. After surgery (3–6 months) 71.7% of patients underwent radiotherapy, 82.1% chemotherapy and 90.7% hormonal therapy.

**Fig. 1:**
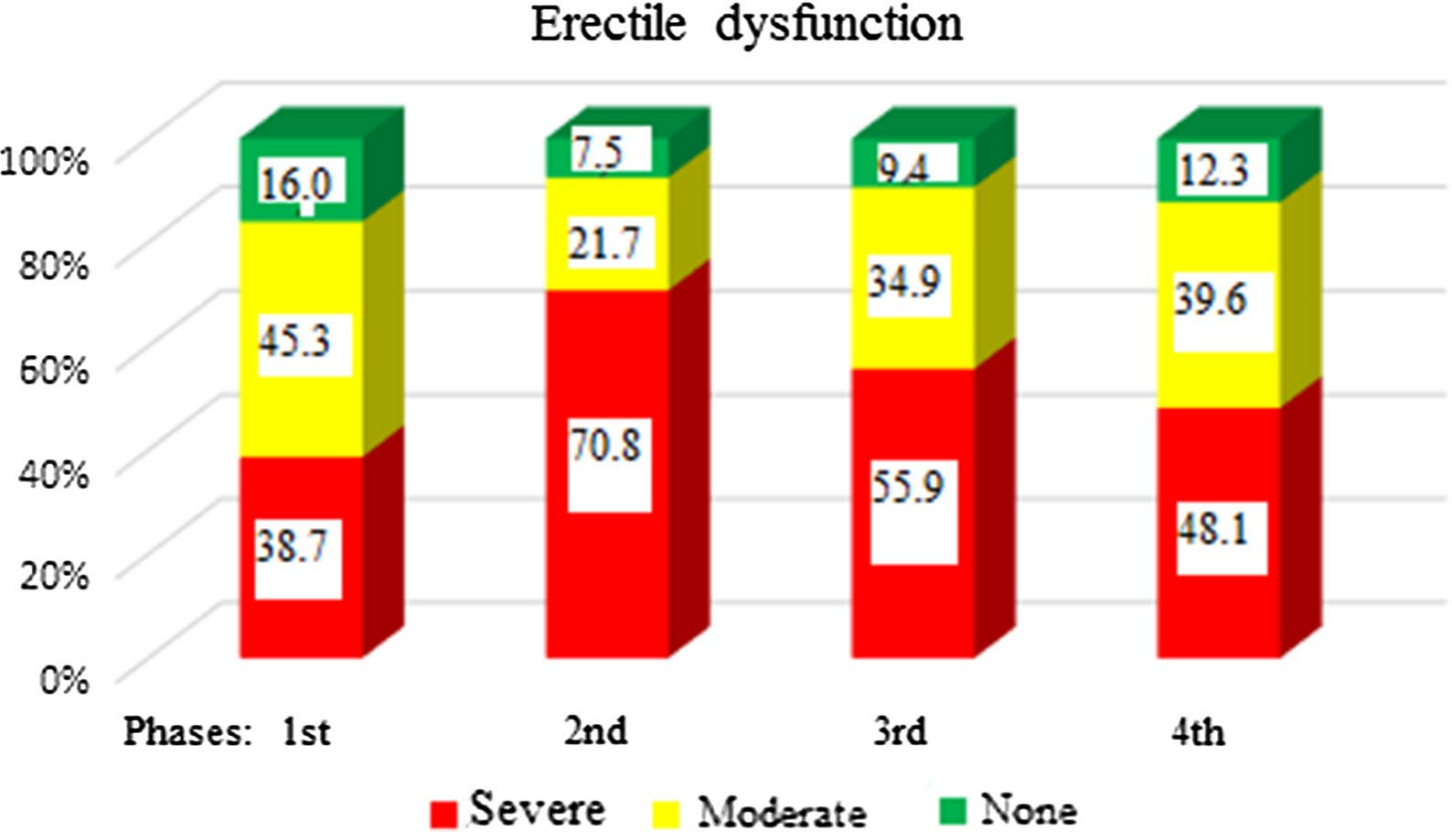
Erectile dysfunction percentages through the four time points of the study

The severe erectile dysfunction rates increased from 38.7% before surgery to 48.1% (25% increase) after surgery, at the end of the follow-up period (p < 0.05) (Fig. 1).

Those with higher educational level exhibited significantly higher levels of erectile function, both before and after surgery, with the differences being particularly pronounced at the 3rd and 4th stage in all subscales (p < 0.01).

Colectomy compared with other interventions had a significant effect on erectile dysfunction, but patients undergoing colectomy/sigmoidectomy had significant erectile dysfunction prior to surgery (p = 0.032).

**Fig. 2:**
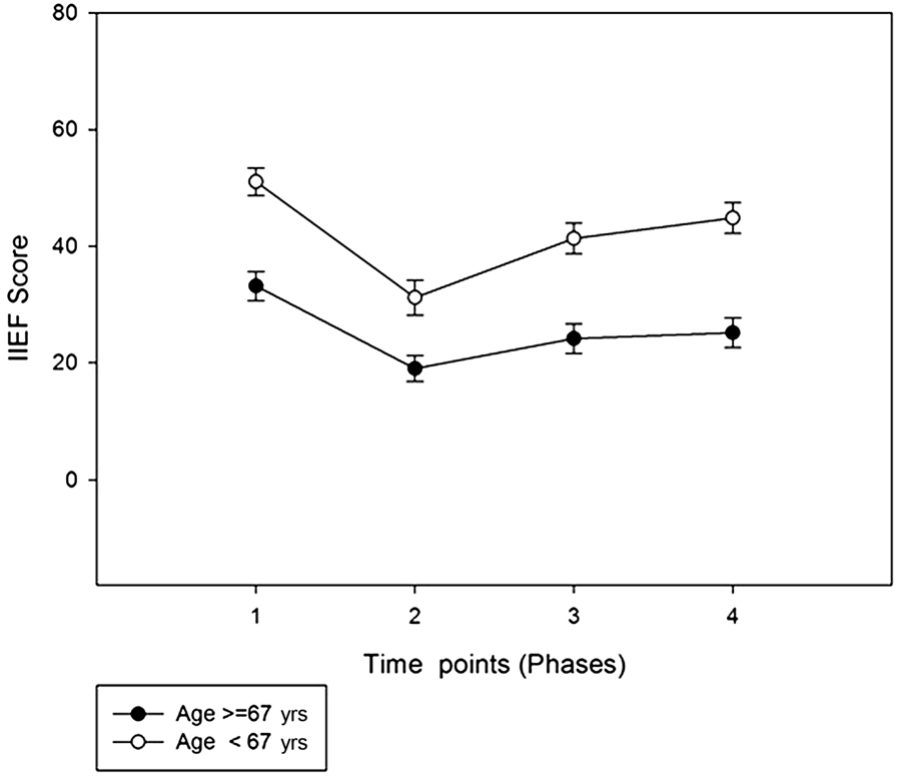
IIEF total score at the four time points of the study depending on patients’ age

Age had a significant effect on erectile dysfunction: Both before surgery and at follow up after that, the differences in patients older than 67 years of age (median age value in the study sample) with those under the age of 67 years were statistically significant in all the subscales of IIEF and in all phases of the study (p < 0.001). The mean value of the overall score was indicative of rather mild erectile function in phase 4 (19.04) in patients under 67 years of age. (Fig. 2)

Regarding chemotherapy after the surgery, no significant difference was found in total IIEF score at all time points (p > 0.200), whereas at phase 4 IIEF score was 39.34 ± 20.97 for those without chemotherapy and 34.70 ± 21.48 for those under chemotherapy (p = 0.394).

In the multivariate analysis model, age emerged as an independent predictor of erectile function (p < 0.001), while marginal correlation was observed with the type of intervention (p = 0.057) (Table 1). When the state “control” or “patient” was entered as a dummy variable along with age (in years) in a linear regression model, both variables were independently associated with IIEF total score at phase 4 (B = − 18.62, p < 0.001 for patient/control group and B = − 0.632, p < 0.001 for age.

**Table 1:** An assessment model of demographic and surgery procedure effect IIEF (Phase 4) total score

Model R^2^= 0.552	Unstandardized coefficients		Standardized coefficient	t	p	95.0% Confidence interval for B	
	B	Std. error	Beta			Lower bound	Upper bound
(constant)	39.221	3.435		11.419	< 0.001	32.408	46.033
Age	− 17.687	3.697	− 0.416	− 4.785	< 0.001	− 25.020	− 10.355
Educational level	6.381	4.032	0.139	1.583	0.117	− 1.616	14.379
Type of surgery	7.162	3.717	0.167	1.927	0.057	− 0.212	14.535

Dependent variable: IIEF (Phase 4) total score

## Discussion

According to the results of this study, age was the most important determinant of the IIEF score, which was aggravated by 25% from the first to the last assessment of the patients. There was a significant difference in erectile function immediately after surgery. Surgical technique and experience remain the main variables that determine the outcome, but other factors that may affect postoperative erectile function include patient age, preoperative sexual function, psychological adaptation to cancer diagnosis and co-morbidity (e.g., diabetes mellitus, hypertension). Other preoperative variables include the stage of the disease, maintenance of neurovascular links, urinary incontinence and adjunctive therapies such as hormone therapy and radiation [8, 9].

Age has a significant effect on the recovery of erectile function in pelvic surgery, particularly post-prostatectomy, since there is a significant reduction in power erection with increasing age. Most series reported a 59–82% potency in patients under 60 years of age versus 36–57% of patients over 60 years of age [8]. Kundu et al. reported that 40–50 year old patients who underwent bilateral nerve retention surgery have almost twice as much chance of regaining erections from patients over 70 years of age. Age is also correlated with the state of erectile function preoperatively, which has been reported to affect the recovery of erectile function [10]. Geary et al [11] and Rabbani et al. [12] reported that pre-operative erectile function has a significant impact on the recovery of spontaneous erections. However, the average follow-up was only 12–24 months. In a study involving a follow-up of 1–3 years and 141 patients, it was found that at 1 year, 113/141 (80%) patients were sexually active, achieving vaginal contact (including medication) and 28 (20%) were sexually inactive. The reasons for sexual inactivity was incontinence (15/28, 53%), loss of interest in sex (10/28, 36%) and libido loss (3/28, 11%, hormonal therapy) [8].

There is no doubt that pelvic surgery does interferes with erectile function and this was evident in the present study, when patients were compared with the controls. Of note, IIEF score in controls was in line with the literature [13, 14] and differed significantly from patients’ IIEF score at phase 1, indicating that even before surgery, pelvic pathology has already an effect on erectile function [15]. However, among patients who underwent surgery, age was the decisive prognostic factor for erectile function. In that context when surgery and age were included in the regression model, both variables were independently associated with erectile function.

Regarding the educational level and its role in the sexual functioning of patients with pelvic surgery, it is likely that the higher educational level contributes to better erectile function, coupled with age and the absence of complications. In the Oudsten et al. [4] study, it was found that sexually active participants were younger, employed and had a higher educational level than the participants who had no sexual activity. Respectively, the findings of Sutsunbuloglu and Vural [16] showed that lower educational level was associated with greater sexual dysfunction.

Regarding the type of surgery in the present study, they were of open type, they were related to different pathological conditions (colectomy, prostatectomy, inguinal hernias etc.); the inguinal hernias accounted for one-third of the interventions, while the number of prostatectomies was small. Indeed, hernia itself and hernia surgery may have an effect on sexual activity, however, the effect expected is smaller compared with other procedures and there may be significant improvement of erectile function. Ertan et al [17] prospectively investigated 34 hernia patients in terms of sexual function before and 3 months after hernia repair using IIEF. They reported that there was a significant improvement in IIEF scores after recovery and that sexual activity was positively affected after surgery. Also, the age and overall health of the patient is another factor affecting rehabilitation time. It is known that younger patients recover faster than the elderly. Also, the older they are, the more co-morbidities exist that do not facilitate rapid recovery and increase the likelihood of complications.

## Limitations

This study has limitations related to sampling and the type of surgery. The sample, although randomized, comes from one hospital, while some patients refused study entry. Perhaps among them there were patients with significant erectile dysfunction and did not want to refer to this sensitive issue. In addition, there was considerable heterogeneity in the type of surgery. Other factors such as the psychological state of patients could also have an impact on erectile function, including various drugs and cancer treatment modalities, that were not extensively investigated in the present study. However, regarding chemotherapy, no significant difference was found between patients who underwent chemotherapy and those who did not. Future research could extensively examine biomedical and psychological factors together with non- modifiable risk factors to investigate the separate contribution of different variables to erectile function.

## Data Availability

A confidentiality agreement with participants prevents us from sharing the data.

## Abbreviations

IIEF: International Index of Erectile Function
APR: abdominoperineal resection
RP: radical prostatectomy
